# Spontaneous vaginoperitoneal fistula as a rare complication of carcinoma ovary: a case report

**DOI:** 10.1186/s43046-021-00099-9

**Published:** 2022-01-03

**Authors:** Tarang Preet Kaur, Sangeeta Bhasin, Asmita M. Rathore

**Affiliations:** 1 Maulana Azad Medical College (MAMC), New Delhi, India; 2 Present Address: All India Institute of Medical Sciences (AIIMS), New Delhi, India

**Keywords:** Vaginoperitoneal fistula, Carcinoma ovary, Spontaneous, Exploratory laparotomy

## Abstract

**Background:**

Spontaneous vaginoperitoneal fistula formation in a case of carcinoma ovary is a very rare occurrence and has never been reported.

**Case presentation:**

A 55-year-old postmenopausal lady presented with complaints of abdominal distention and mass coming out of the vagina for the last 10 days. On examination, she had tense ascites, uterovaginal prolapse and hard, fixed mass felt anteriorly on per-rectal examination. Biochemical investigations and radiological imaging suggested advanced stage ovarian neoplasm. She was planned for neoadjuvant chemotherapy. During the second cycle of chemotherapy, she developed spontaneous vaginoperitoneal fistula which was confirmed on exploratory laparotomy where interval debulking surgery was performed in collaboration with gastro-surgeons on a semi-emergency basis. The postoperative course was uneventful.

**Conclusion:**

Spontaneous vaginoperitoneal fistula is a rare complication and should be kept in mind while managing advanced ovarian neoplasm.

## Background

A fistulous communication between the vagina and the bladder, urethra, ureter or rectum following an abdominopelvic surgery is a common cause of continuous vaginal discharge or urinary leakage. However, spontaneous development of a vaginoperitoneal fistula in a case of carcinoma ovary is a very rare occurrence and has never been reported. Here, we describe a case of FIGO stage IV B ovarian cancer with spontaneous vaginoperitoneal fistula.

## Case presentation

**Fig. 1:**
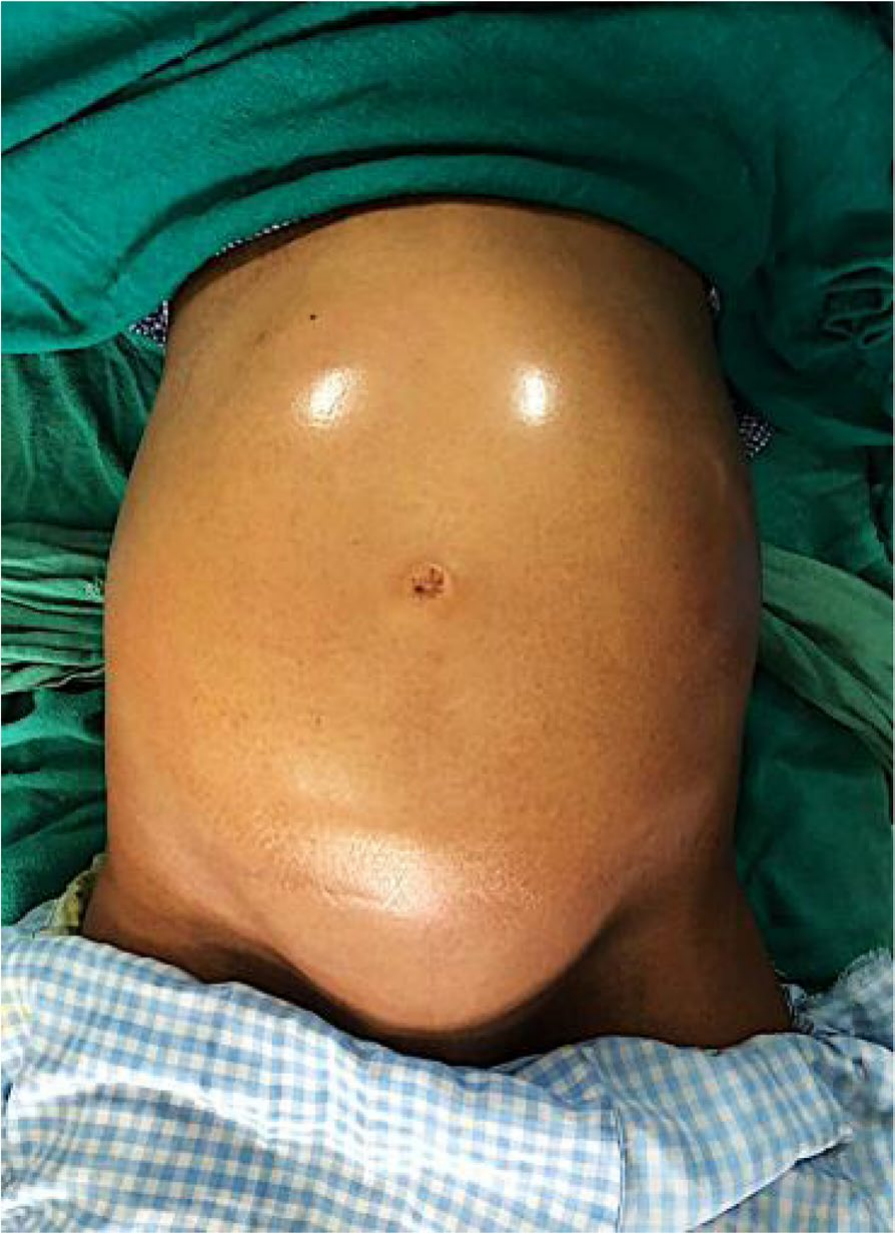
Abdominal distention on initial presentation due to mass and tense ascites

A 55-year-old, frail-looking multiparous postmenopausal lady presented with the chief complaints of anorexia and easy fatigability for one month and abdominal distention, decreased urine output and constipation for the last 10 days. She also complained of mass coming out of the vagina for the last 10 days. There was no significant medical, family, psycho-social or past history. On clinical examination, except for the presence of bilateral pedal oedema, her general physical examination was unremarkable. On abdominal examination, she had tense ascites and no abdominal mass could be appreciated. Second-degree cervical descent, cystocele and rectocele were present on local examination (Fig. 1). On per-speculum examination, the cervix and vagina appeared healthy. On pelvic examination, all the fornices were full due to tense ascites and exact uterine size could not be made out. On per-rectal examination, rectal mucosa was free but a hard and fixed mass was felt anteriorly which was high-up in location.

All her routine blood investigations were within normal range except for decreased proteins [serum total proteins = 3.2 gm/dl (6–8.3 gm/dl), serum albumin = 1.8 gm/dl (3.5–5.5 gm/dl)] and raised serum lactate dehydrogenase (1068 U/ml, normal = 100–190 U/L). Biochemical investigations revealed raised levels of tumour markers such as cancer antigen-125 (CA-125) = 225.0 mIU/L (normal = 0–35 mIU/L) and carcinoembryonic antigen = 146.5 ng/ml (normal ≤ 3.4 ng/ml). The abdominal ultrasound (USG) showed the presence of gross ascites and a 10 × 9 × 8 cm solid cystic mass with increased vascularity and multiple septae in the lower abdomen. Uterus measured 6 × 2.5 × 1.5 cm with normal myometrial echotexture. Bilateral ovaries were not visualised. Contrast-enhanced computed tomography (CECT) abdomen revealed the presence of gross ascites with 10.2 × 10.7 × 12.5 cm solid-cystic mass arising from bilateral ovaries. It was extending to the pouch of Douglas (POD), abutting the rectum and sigmoid posteriorly and pelvic wall laterally with maintained fat planes. Hypodense soft tissue deposits likely metastasis were also reported in a subcapsular location in the liver (2.5 × 2 cm), pancreas (2.8 × 1.8 cm) and at the gastro-oesophageal junction (1 × 1 cm). A radiological diagnosis of ovarian neoplasm with metastasis was given. An ascitic fluid examination done for malignant cytology was suggestive of mucin secreting neoplasm. USG guided fine needle aspiration cytology, from the mass was reported as adenocarcinoma. Upper Gastrointestinal and lower Gastrointestinal endoscopies were unremarkable. Her Risk of malignancy index score was 2025; suggestive of malignancy. Using the International Ovarian Tumour Analysis ADNEX model various risks calculated were 42.8% for benign, 57.2% for malignant and 41% for borderline [1].

**Fig. 2:**
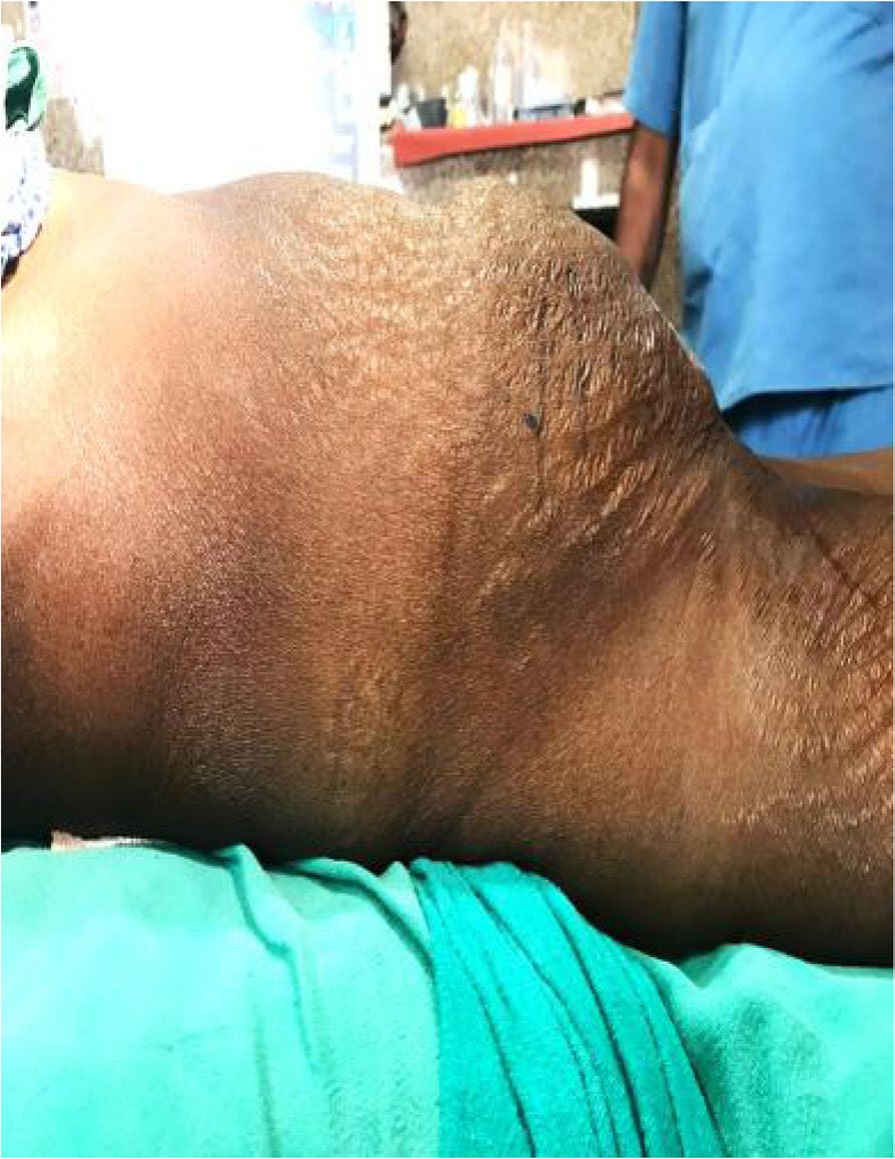
Ascites resolved post-vaginoperitoneal fistula formation

**Fig. 3:**
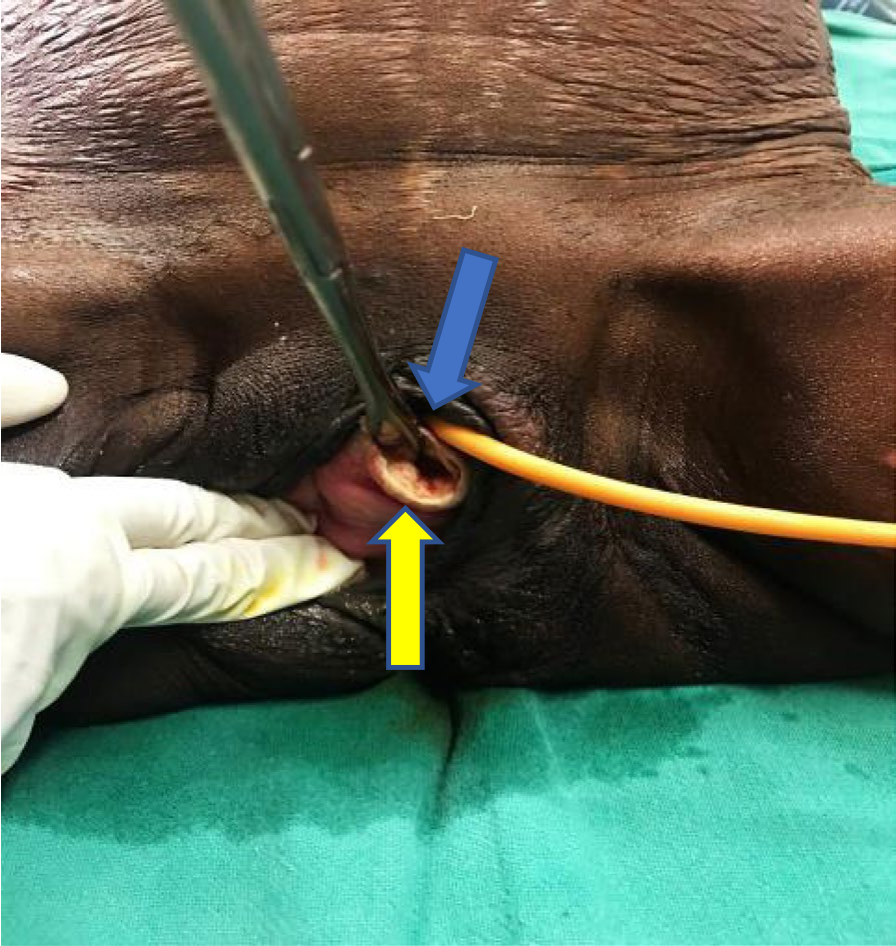
Local examination of vaginoperitoneal fistula (blue arrow depicts that cervix is located anterior to the fistula. Yellow arrow shows rent in posterior vaginal wall 3 × 3 cm)

A preoperative diagnosis of FIGO stage IV ovarian carcinoma was made and she was planned for neoadjuvant chemotherapy followed by interval debulking and adjuvant chemotherapy. She received her first cycle of paclitaxel and carboplatin-based chemotherapy uneventfully. During her second cycle, while still admitted in the hospital, she reported an episode of sudden, unprovoked, spontaneous, painless gush of fluid coming out through her vagina while she was ambulating. On examination, she was hemodynamically stable and a firm to hard mass with restricted mobility could now be appreciated in the lower abdomen (Fig. 2). On per speculum examination, a defect measuring 3 × 3 cm was seen in the posterior vaginal wall 1 cm from the cervical os (Fig. 3). It had ragged and necrotic margins and fluid with mucus flakes was seen draining out through it. These findings were confirmed on vaginal examination.

**Fig. 4:**
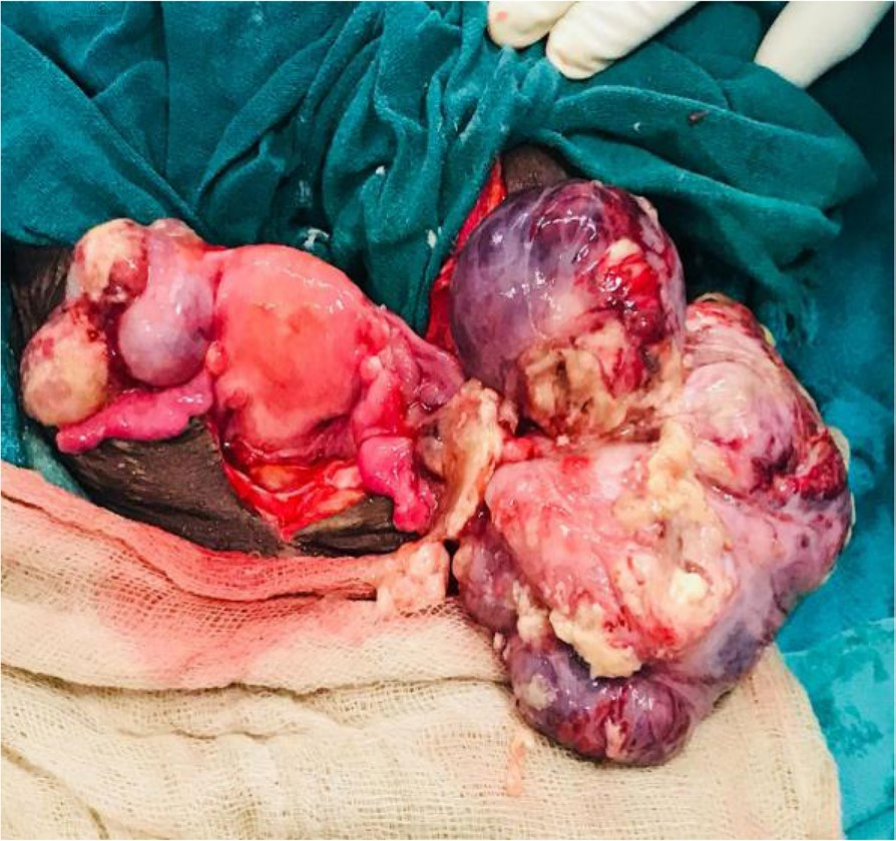
Bilateral adnexal masses showing growth. Right and left adnexal masses measuring 9 × 6 × 5 cm each

**Fig. 5:**
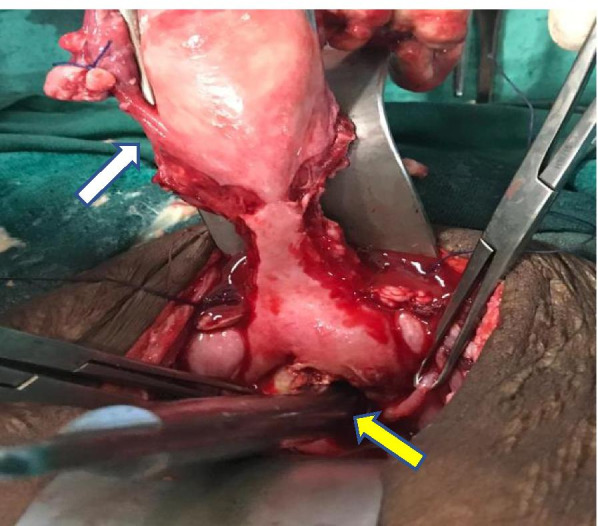
Posterior vaginal wall showing rent 3 × 3 cm (yellow arrow) and uterus (white arrow)

The patient was now planned for an exploratory laparotomy on a semi-emergency basis in collaboration with gastro-surgeons. Per-operatively, there was minimal ascites along with left and right ovarian masses, irregular, solid-cystic and each measuring 9 × 6 × 4 cm. There were multiple metastatic omental deposits, the largest measuring 1 cm (Fig. 4). No tumour deposit was felt on the liver surface, stomach, spleen, colon, small intestine, pancreas, paracolic gutters or pouch of Douglas. No retroperitoneal or pelvic lymph nodes were palpated. A defect with necrotic margins was confirmed in the posterior vaginal wall which measured 3 × 3 cm (Fig. 5). A total abdominal hysterectomy with bilateral salpingo-oophorectomy with omentectomy with appendicectomy was performed. The posterior vaginal wall rent was repaired in two layers. An intra-abdominal drain was put in and left for 15 days postoperatively in order to avoid pressure impact on the repaired area in case of recurrence of ascites. The patient tolerated the procedure well.

### Post-operative course

Mild leak per-vaginum continued postoperatively and stopped on the 3rd postoperative day. Stitch removal was done on day 10. The final histopathology report read as bilateral adnexal masses showing mucinous adenocarcinoma with tumour cells infiltrating the capsule along with multiple foci of tumour deposits in the omentum. No evidence of malignancy was seen in the specimen of uterus, cervix, fallopian tubes, appendix or the excisional biopsy taken from the margin of the defect in the posterior vaginal wall. Adjuvant chemotherapy with paclitaxel and carboplatin was started 3 weeks postoperatively and 4 cycles were given. Post chemotherapy, the patient appeared clinically well and radiologically tumour free with CA 125 level of 38 IU/ml for a follow-up period of 8 months. She is currently under follow up. There has been no adverse or unanticipated events till the date of follow-up.

## Discussion

Vaginoperitoneal fistula is a rare entity in itself and its spontaneous formation in a patient of carcinoma ovary has never been reported [2–6]. A computerized literature search was carried out using Medlars database supplied by the US National Library of Medicine and no similar report was found between the time period 1986–2020. Terms searched included vaginoperitoneal fistula carcinoma ovary, prolapse vaginoperitoneal fistula, malignant ascites vaginoperitoneal fistula, vaginoperitoneal fistula chemotherapy.

We found few case reports of vaginoperitoneal fistula, out of which only two developed spontaneously, the rest developed after hysterectomy (abdominal or vaginal). Among the spontaneous vaginoperitoneal fistulas, one was seen in a patient of carcinoma cervix stage IV and the other developed in a case of ruptured appendicitis related pelvic abscess [2, 6]. Among the post hysterectomy fistulas, the most commonly reported one was that seen after fallopian tube prolapse occurring after hysterectomy [5]. Others had resulted after some form of trauma to the vault after hysterectomy [3, 4]. An interesting case was reported by Singla et al. in which a young girl presented with pneumoperitoneum and a vaginoperitoneal fistula, 10 weeks after she had undergone abdominal hysterectomy and bilateral salpingo-oophorectomy for carcinoma ovary, the fistula most likely resulted after her first coital act post-surgery [3]. Vaginoperitoneal fistula following jacuzzi usage after hysterectomy and after vaginal vault fixation of peritoneal dialysis catheter has also been reported [4, 11].

We hypothesised various causes that could have led to the development of spontaneous vaginoperitoneal fistula in our patient. Firstly, malignancy per se could have contributed to it. A metastatic deposit in the POD could have progressed, eaten its way through the vagina and got flushed out along with the gush of ascitic fluid or it could have undergone lysis under the effect of chemotherapy, thus giving way and leading to the formation of a vaginoperitoneal fistula. Secondly, it could have been a rare complication of paclitaxel and carboplatin chemotherapy. Ischemic colitis and neutropenia-related necrotizing enterocolitis leading to bowel perforation have been reported as an uncommon complication with this therapy [7–12]. Around 10 such cases have been reported out of which 9 were in relation to ovarian cancer and most of them had this complication after the first chemotherapy cycle. As an extrapolation, the same mechanism of necrosis and perforation could have led to the vaginoperitoneal fistula formation in our patient. Thirdly, it could be ascribed to the intense pressure exerted by the gross ascites on the weak postmenopausal prolapsed tissues of this immunocompromised patient.

Spontaneous development of a vaginoperitoneal fistula in our patient of stage IV carcinoma ovary after receiving one cycle of neoadjuvant chemotherapy left us in a state of dilemma as to the course of further management. We could have either let her continue and complete her neoadjuvant chemotherapy so that chances of optimal debulking during surgery would increase or we could have taken her up for immediate exploratory laparotomy so that chances of her developing ascending peritonitis through the open communication would decrease. Literature search did not help us as such a case has never been reported. We chose to operate upon her on a semi-emergency basis to prevent any form of ascending infection thus leading to peritonitis and sepsis.

## Conclusions

Though vaginoperitoneal fistulas following hysterectomy for benign or malignant conditions occur, the development of a spontaneous vaginoperitoneal fistula in a patient of carcinoma ovary is being reported for the first time. This rare complication should be kept in mind while treating patients of advanced ovarian malignancy and should be reported as it will help in formulating standard guidelines for its management.

## Data Availability

All data generated or analysed during this study are included in this published article.

## Abbreviations

CA-125: Carcinoembryonic antigen
CECT: Contrast-enhanced computed tomography
POD: Pouch of Douglas
USG: Ultrasonogram
